# A Rare Case of Left Ventricular Thrombus in a Normal Heart in a Patient With Factor V Leiden Disease

**DOI:** 10.7759/cureus.67104

**Published:** 2024-08-18

**Authors:** Rupesh Kshetri, Pragya Pathak, Prasanna Sugathan

**Affiliations:** 1 Internal Medicine, University of Iowa Hospitals and Clinics, Iowa City, USA; 2 General Medicine, Sirthauli Hospital, Dudhauli, NPL; 3 Cardiology, Imperial Health, Moss Bluff, USA

**Keywords:** factor v leiden, anticoagulation, ejection fraction, thrombosis, left ventricle

## Abstract

Left ventricular thrombus (LVT) is mostly associated with anterior wall myocardial infarction and reduced ejection fraction. It can also be associated with cardiomyopathy, myocarditis, and hypercoagulable states such as cancer, antiphospholipid syndrome, and protein C or protein S deficiency. Factor V Leiden (FVL) disease is one of the hypercoagulable states where mutant factor V is insensitive to natural anticoagulation factor protein C, and FVL disease increases the risk of peripheral thromboembolism such as pulmonary embolism (PE) and deep vein thrombosis (DVT). We report a 60-year-old female patient with a history of heterozygous factor V Leiden and a remote history of deep vein thrombosis who presented with left-sided weakness and intermittent chest pain. Computed tomography (CT) of the brain ruled out stroke, electrocardiogram (EKG) showed sinus rhythm and some new T-wave inversion, and troponin was mildly elevated. Other laboratory results were unremarkable. A transthoracic echocardiogram showed a left ventricular mass with left ventricular outflow tract (LVOT) obstruction in systole with normal systolic and diastolic function and no wall motion abnormalities. Emergent surgery proved to be a thrombus. The learning objectives of our case are that a normal-sized and functional left ventricle does not preclude left ventricular thrombosis, long-term anticoagulation therapy in patients with factor V Leiden and a first episode of thromboembolism with additional risk factors may prevent further serious thromboembolic event, and timely diagnosis and treatment of cardiac thrombosis may reduce morbidity and mortality.

## Introduction

Left ventricular thrombus (LVT) is a potentially life-threatening complication of myocardial infarction and left ventricular dysfunction. Left ventricular thrombus is mostly associated with anterior wall myocardial infarction with reduced ejection fraction and is also seen with cardiomyopathy, myocarditis, protein C or S deficiencies, cancers, antiphospholipid syndrome, and muscular dystrophies [1]. There are case reports of left ventricular thrombus without ventricular dysfunction in hypercoagulable states [2-4]. Factor V Leiden (FVL) usually causes deep vein thrombosis (DVT) and pulmonary embolism (PE). We found a case of factor V Leiden with right ventricular thrombus with no ventricular dysfunction [5] in a PubMed search. However, our patient with factor V Leiden has a thrombus in a normal-sized and functional left ventricle.

## Case presentation

A 60-year-old female with a history of heterozygous factor V Leiden (FVL) and deep vein thrombosis (DVT) in her right leg was on direct oral anticoagulant for a year, but this was discontinued due to economic reasons. She presented to her primary care physician with a complaint of left-sided weakness intermittently for a couple of days. She also reported symptoms of intermittent tingling and numbness, intermittent shortness of breath with exertion, palpitations, and occasional chest pain not associated with activity for the last three months. She was admitted for further evaluation.

On examination, the patient's vital signs were stable with a blood pressure of 98/59 mmHg and a heart rate of 85 beats per minute. Her heart had a regular rate and rhythm with a soft systolic murmur at the bases, her lungs were clear to auscultation, and the rest of the examination, including neurological examination, was unremarkable.

Past medical and surgical history included prior smoking (quit about a year ago), hypertension, hyperlipidemia, type 2 diabetes mellitus, anxiety, adjustment disorder, obstructive sleep apnea with a BMI of 41 kg/m^2^ on continuous positive airway pressure therapy, a heart catheterization 6-7 years ago with no intervention, up-to-date malignancy screening with no malignancy, appendectomy, cholecystectomy, hysterectomy, and urinary bladder repair.

The patient had a family history of DVT in her mother when she was bedridden due to lung cancer. However, there was no family history of FVL or unprovoked thrombosis. Her father had coronary artery disease (CAD) in his 50s, liver cirrhosis, and diabetes mellitus. Her brother had lung cancer.

Investigations

Blood tests revealed normal electrolytes, renal function, liver function, lipid panel, coagulation profile, and hemoglobin and hematocrit levels. Glycated hemoglobin (A1c) was elevated to 9.8%. She initially had 15 ng/L high-sensitivity troponin, followed by 14 ng/L (normal for females: 0-14 ng/L).

**Figure 1:**
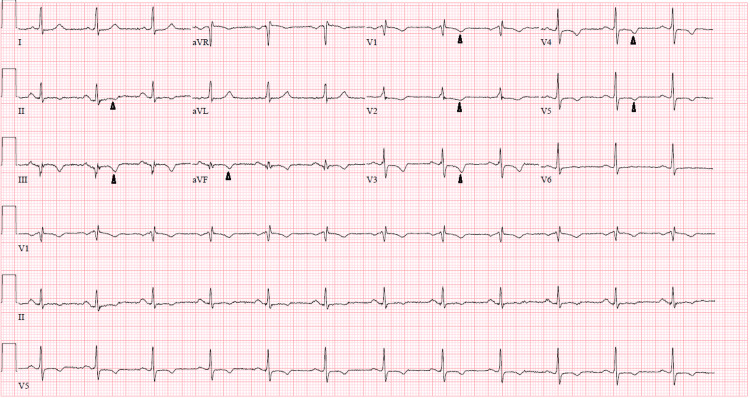
EKG showing sinus rhythm and T-wave inversion (arrowhead) EKG: electrocardiogram

A computed tomography (CT) image of the brain showed no acute ischemia, infarct, or mass; the lower extremity duplex imaging was negative for DVT. Electrocardiogram (EKG) revealed sinus rhythm with incomplete right bundle branch block (RBBB) and T-wave inversion in leads II, III, aVF, and V3-V5, consistent with inferior and anterolateral ischemia (Figure 1). Two years prior, the EKG showed T-wave inversion in leads V1-V3 with RBBB.

**Figure 2:**
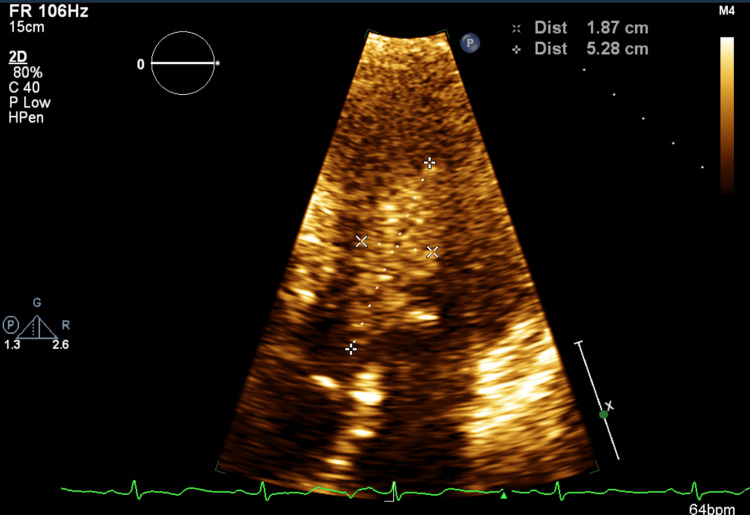
LV thrombus without contrast (longitudinal view) LV: left ventricular

**Figure 3:**
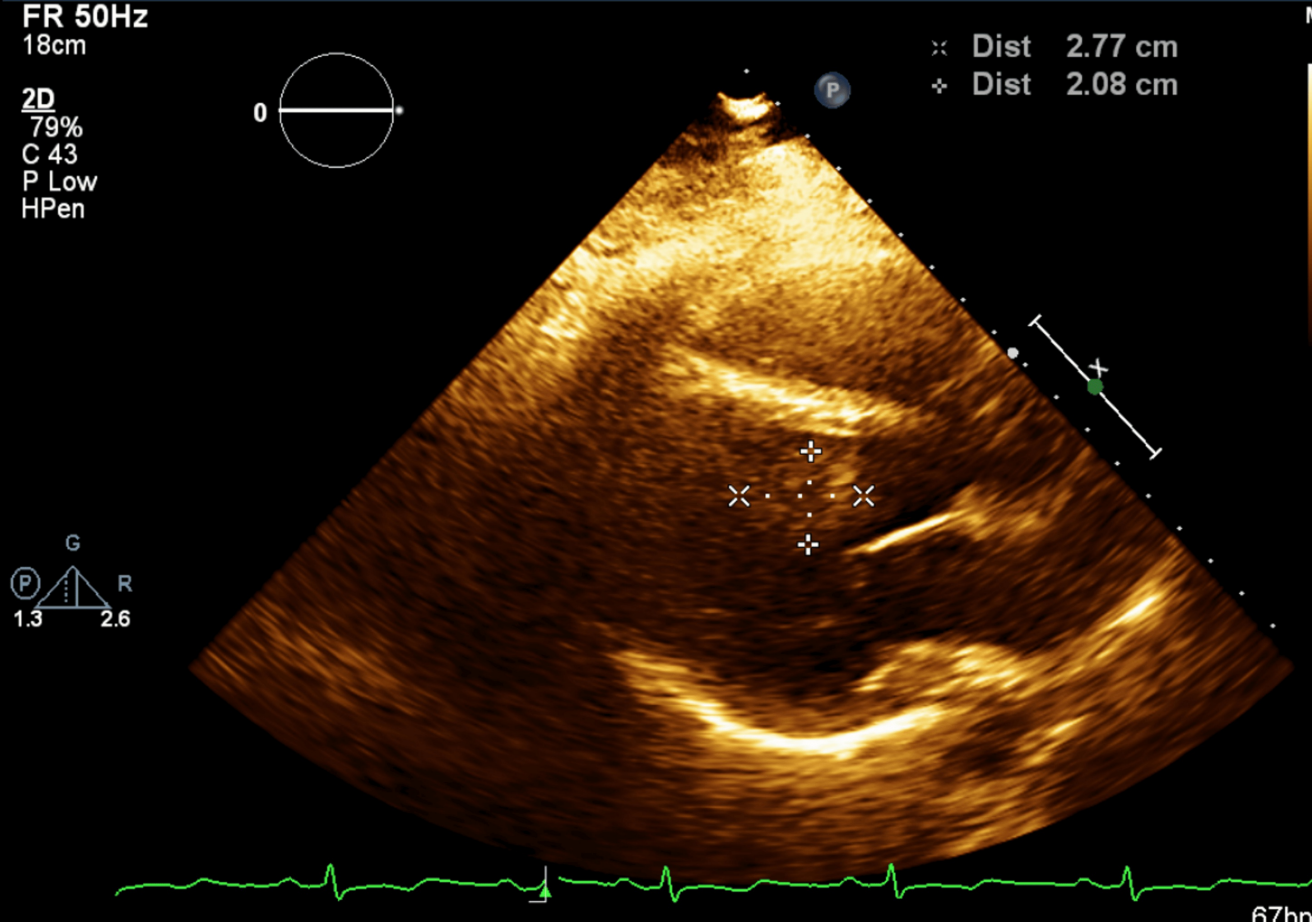
LV thrombus without contrast (horizontal view) LV: left ventricular

**Figure 4:**
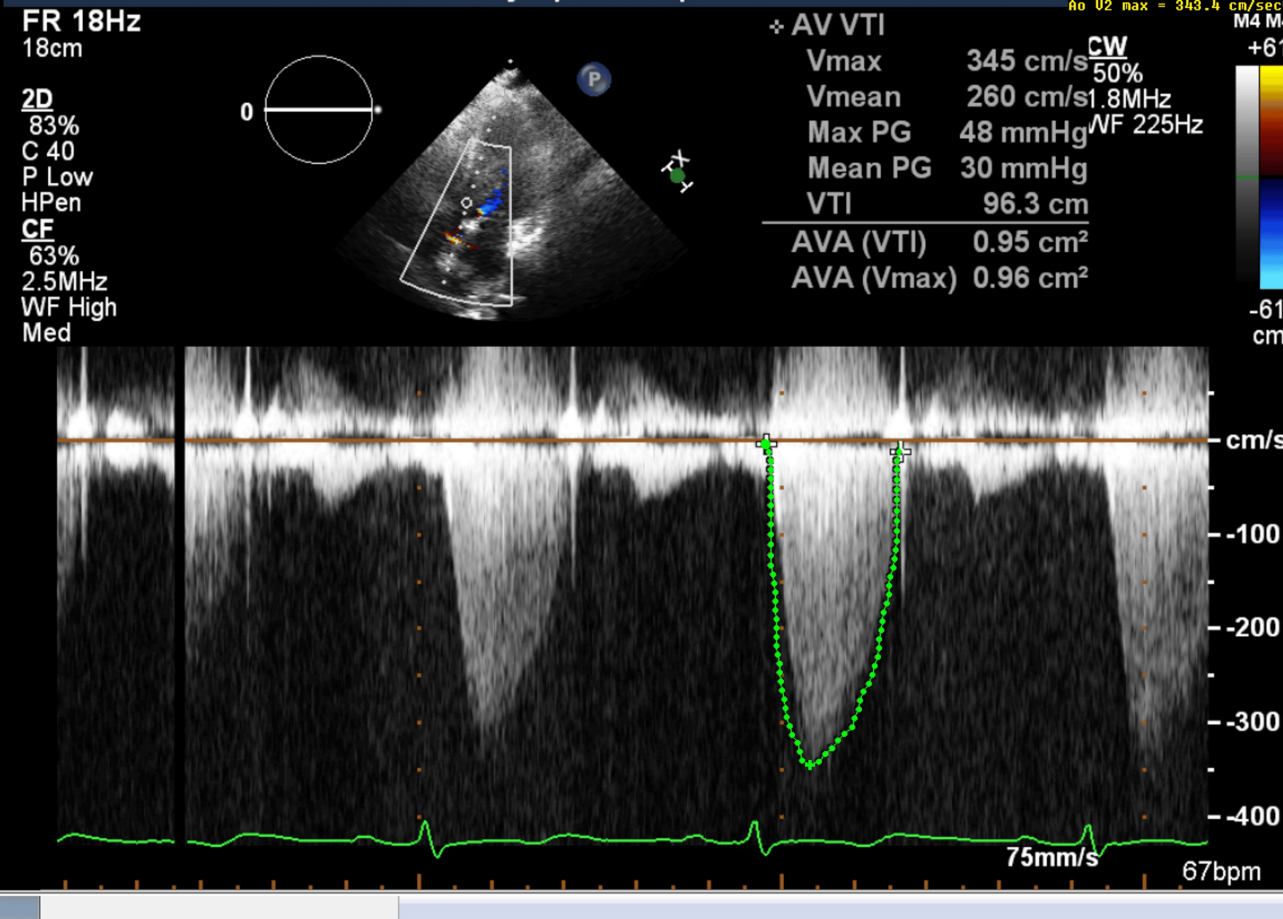
Doppler echo showing LVOT obstruction LVOT: left ventricular outflow tract

A two-dimensional echocardiogram showed normal biventricular systolic and diastolic functions with a left ventricular ejection fraction (LVEF) of 60% and normal valve function. A mobile 5.9 cm × 1.9 cm oblong, irregularly bordered, homogeneous-appearing tissue density mass was present in the left ventricle attached to the mid-septum and apical inferior wall, partially obstructing the left ventricular outflow tract (LVOT) in systole. The mass was not opacified by echocardiography contrast, suggestive of a thrombus (Figures 2-4).

Management

The differential diagnosis at admission was stroke versus coronary artery disease. However, the transthoracic echocardiogram found a mass in the left ventricle, so our differential diagnosis changed to left ventricular thrombus versus cardiac tumor.

A transesophageal echocardiogram (TEE) was planned, but given the high risk of the procedure, the patient was transferred to a tertiary center where she underwent urgent cardiac surgery with removal of the mass after confirmation by TEE. It was a solid, firm mass attached to the intraventricular septum. The mass was removed via the aortic valve route with aortotomy in two settings since post-procedural TEE showed some residual material attached to the septum. Pathology report of mass proved a large, organized thrombus.

On the second postoperative day, a left brachial artery thrombus on the A-line site was discovered that was removed by open brachial thrombectomy. She was started on heparin infusion and warfarin after surgery. The rest of her hospital stay was uneventful, and she was discharged in seven days.

The patient was discharged with warfarin with a plan to continue lifelong anticoagulation. After six weeks, she followed a cardiologist and changed warfarin to direct-acting oral anticoagulation.

## Discussion

Left ventricular thrombus is a potentially life-threatening complication of myocardial infarction and left ventricular dysfunction [6]. There are case reports of left ventricular thrombus without ventricular dysfunction in hypercoagulable conditions [2-4]. Factor V Leiden usually causes DVT and PE. Right ventricular thrombus with FVL and normal ventricular function has been reported [5]. This is a rare case of a thrombus in a structurally and functionally normal left ventricle and FVL.

Factor V Leiden is mutant factor V that is insensitive to activated natural anticoagulation factor protein C, increasing the risk of venous thromboembolism (VTE) [5]. Factor V Leiden is the most common inherited hypercoagulable state and is mostly associated with peripheral venous thrombosis and PE [7]. Around 5% of Caucasians have heterozygous FVL, and up to 30% of patients presenting with DVT or PE suffer from FVL [5,7]. Approximately one in 1,000 general patients develop DVT or PE each year [7]. Heterozygous FVL increases the risk of thromboembolism 5- to sevenfold, and homozygous FVL increases the risk 25- to 50-fold [7]. The odds of recurrent venous thromboembolism (VTE) in a heterozygous FVL patient after a first episode of VTE following discontinuation of anticoagulation is 1.4 [8]. Although FVL is proven to cause VTE, there is controversy regarding FVL causing arterial/cardiac thrombosis. A review article by Kujovich [9] cited different studies to explain that the risk of VTE and cardiovascular events is increased in patients with FVL and having other circumstantial/environmental risk factors such as age, obesity, smoking, travel, hypertension, and diabetes mellitus. Celik et al. [10] could not establish FVL as a risk factor for LV thrombus in acute myocardial infarction patients. The study has not explored the presence of other risk factors in addition to FVL. Long-term anticoagulation for FVL after the first episode of VTE is usually not recommended unless the patient has additional risk factors for thromboembolism [7]. Our patient had two additional risk factors for thromboembolism: obesity and type 2 diabetes mellitus. Long-term anticoagulation was stopped by our patient due to cost concerns. Treatment options for ventricular thrombus are medical (anticoagulation and thrombolysis) and surgery with anticoagulation [1]. Our patient was treated with surgery followed by anticoagulation because of the large size of the thrombus, high risk of having systemic embolization (mobile and protruding thrombi [1]), and intermittent left ventricular outlet obstruction.

Virchow's triad explains that the pathogenesis of thrombus formation includes disturbance of blood flow (stasis or turbulence), hypercoagulability, and endothelial injury/dysfunction. Hypothetically, even in hypercoagulable states, blood should not clot in the normal functioning heart due to the active flow of blood. Left ventricular thrombi are mostly associated with cardiac abnormalities such as anterior wall myocardial infarction with reduced ejection fraction, cardiac aneurysm, cardiomyopathy, myocarditis, and the use of cardiac devices or hypercoagulable states such as protein C or S deficiencies, malignancies, antiphospholipid syndrome, or pheochromocytoma. Due to the serious complications of left ventricular thrombosis such as CAD, stroke, and systemic thromboembolism, it requires urgent treatment [6]. We can hypothesize that our patient might have transient takotsubo cardiomyopathy, which might have caused the thrombus formation in this high-risk patient with FVL, obesity, diabetes, and a long history of smoking. However, an explanation of thrombus in the normal left ventricle may need further study.

## Conclusions

Cardiac thrombosis in a normally functioning heart is not common, but if it is not diagnosed and not treated in time, it can cause high mortality and morbidity. High-risk patients with factor V Leiden along with other risk factors can have left ventricular thrombosis even in a normal functioning heart. Preventing the condition from occurring with appropriate anticoagulation is a better option for management.
